# Multi-metal adsorption behavior of PP and PVC microplastics: polymer-dependent interactions and mechanisms

**DOI:** 10.1007/s10653-026-03316-3

**Published:** 2026-06-30

**Authors:** İlknur Demirtaş, Parisa Akbari Dana, Zehra Yiğit Avdan, Kadir Gedik

**Affiliations:** 1 Department of Environmental Engineering, Eskişehir Technical University, 26555 Eskişehir, Turkey; 2 Environmental Research Center (ÇEVMER), Eskişehir Technical University, 26555 Eskişehir, Turkey

**Keywords:** Aquatic pollution, Polypropylene, Polyvinyl chloride, Adsorption kinetics, Isotherm modeling, Sorption mechanism

## Abstract

Microplastics (MPs) can act as carriers for co-occurring contaminants in aquatic environments, potentially influencing the mobility, partitioning, and environmental fate of trace metals. In this study, the adsorption behavior of Mn, Ni, Co, and Ba onto pristine MP particles, specifically polypropylene (PP) and polyvinyl chloride (PVC), was investigated in a multi-metal system. MP-metal interactions were characterized using complementary surface, spectroscopic, microscopic, and elemental analyses (BET, FTIR-ATR, SEM-EDX, ICP-OES). The results indicate generally higher adsorption for Co, Ni, and Mn compared to Ba for both MPs, with equilibrium adsorption capacities ranging from 0.011 to 0.032 mg/g for PVC and 0.016 to 0.041 mg/g for PP after 48 h. Kinetic analyses indicated that adsorption onto PP was best described by the pseudo-first-order model, whereas PVC followed the Elovich model, suggesting differences in surface heterogeneity and interaction behavior. Equilibrium data were well fitted the Langmuir model for both polymers, reflecting apparent monolayer-like behavior in the multi-metal system rather than true monolayer adsorption on heterogeneous MP surfaces. Surface analyses revealed polymer-dependent metal distributions and changes in characteristic functional group regions, which were more pronounced for PP. These findings provide insight into the factors governing metal adsorption onto MPs under multi-metal conditions and highlight their potential role as supplementary carriers influencing metal distribution in aquatic systems.

**Supplementary Information:**

The online version contains supplementary material available at 10.1007/s10653-026-03316-3.

## Introduction

Anthropogenic chemicals or materials represent a significant global environmental challenge, driven by increasing production, consumption, and accumulation of plastic waste in various environmental compartments, especially in aquatic ecosystems (Al Alwan et al., 2024; Bao et al., 2024; Dana et al., 2024; Zhang et al., 2021). The widespread use and limited recycling of commodity plastics such as polypropylene (PP) and polyvinyl chloride (PVC) have contributed to the accumulation of polymeric pollutants, which undergo fragmentation into microplastics (MPs; 1 µm–5 mm) through abiotic and biotic processes (PlasticsEurope, 2024; Campanale et al., 2020; Crawford & Quinn, 2017). In this context, given the geo-accessibility and bio-accessibility of pollutants, MPs can interact with surrounding media and act as passive samplers and potential vectors for inorganic and organic contaminants, raising concerns about their environmental and health implications (Biswas et al., 2024). A bibliometric analysis of the literature indicates that a substantial majority of MP-related studies (over 96%) have focused on marine and aquatic ecosystems (Xu et al., 2020). However, most of these studies have primarily investigated the occurrence and distribution of MPs, while comparatively fewer studies have addressed their interactions with coexisting chemical contaminants. In particular, data on the coexistence and interactive behavior of multiple metal ions in the presence of MPs remain limited (Bi et al., 2024). This gap is particularly important, as natural aquatic systems or wastewater-impacted systems typically contain multiple coexisting metal ions, making such interactions environmentally relevant. In aquatic environments, these particles may influence the geochemical cycling, partitioning, and distribution of trace metals by providing additional reactive surfaces for adsorption, with indirect implications for metal mobility.

The presence of MPs in the environment has been associated with a range of environmental and health concerns, particularly in relation to their potential interaction with heavy metals. A substantial body of evidence indicates that such interactions may result in adverse effects on water and soil ecosystems, disrupt the growth and activity of various organisms, and pose a considerable risk to human health through food chain transfer (Bowley et al., 2021; Gong et al., 2023; Palmer & Herat, 2021; Ziajahromi et al., 2016). The capacity of MPs to adsorb heavy metals varies depending on both the type of metal ion and the characteristics of the MPs, which are largely governed by differences in their physicochemical properties (Han et al., 2021). In addition, environmental factors such as salinity, pH, and metal concentration play critical roles in shaping adsorption behavior (Barus et al., 2021, 2023; Vrinda et al., 2023). Heavy metal ions can interact with MP surfaces through several mechanisms, primarily electrostatic attraction between cationic metal species and negatively charged surface functional groups, ion exchange, and surface complexation via coordinate covalent bonding with electron-donating groups such as carboxyl, hydroxyl, and carbonyl moieties (Bi et al., 2024; Cao et al., 2021; Song et al., 2022). The transport-related interactions (i.e., the potential role of MPs as contaminant carriers) between heavy metals and MPs have therefore become a focal point of extensive research in recent years (Adeleye et al., 2024; Binda et al., 2021; Narwal et al., 2024; Tenea et al., 2024). Several studies have investigated the adsorption characteristics of various MP types (PA, PE, PP, PVC, PS, PET, etc.) in relation to a range of heavy metals, including Cd, Cu, Pb, Cr, Zn, As, and Ni (Chen et al., 2022; Fan et al., 2021a; Hu et al., 2020; Li et al., 2020; Shao et al., 2023; Wang et al., 2017, 2022; Zou et al., 2020). However, studies addressing multi-metal adsorption that originates from industrial inputs and geogenic sources remain relatively scarce in the extant literature (Barus et al., 2023; Chen et al., 2023; Fan et al., 2021a; Gao et al., 2019; Huang et al., 2020; Liu et al., 2021b; Wang et al., 2022). Existing findings suggest that the presence of multiple metal ions (Pb, Cu, Zn, Cd) generally reduces overall adsorption capacities compared to single-metal systems, underscoring competition for limited adsorption sites on MPs such as PP, PVC, PS, PET (Fan et al., 2021a; Gao et al., 2019; Wang et al., 2022).

A variety of kinetic, isotherm, and thermodynamic models are commonly employed to describe adsorption behavior and provide insight into potential interaction mechanisms (Bian et al., 2024; Öz et al., 2019; Raji et al., 2023). Nevertheless, multi-component adsorption and modeling studies remain underrepresented in the MP literature. Consequently, both transport-related processes and multi-metal interaction studies require a more comprehensive examination of how heavy metals influence one another and how sorbent-sorbate associations evolve under combined interaction scenarios. In this context, the present study investigates the adsorption behavior of heavy metal ions (Mn, Ba, Ni, Co) onto pristine PP and PVC microplastics in a multi-metal system. These metals were selected to represent environmentally relevant trace elements originating from both geogenic and anthropogenic sources, with contrasting physicochemical properties (e.g., ionic radius, hydration behavior, and hydrolysis tendency). PP and PVC were chosen as representative commodity polymers with distinct surface characteristics (non-polar vs. polar) and high environmental prevalence. This selection enables the evaluation of polymer-dependent adsorption behavior under multi-metal conditions and contributes to a better understanding of metal-MP interactions and their potential relevance to trace metal fate and transport in aquatic systems. Although the present study does not fully replicate complex natural aquatic matrices, Milli-Q water was used to isolate intrinsic metal-MP interactions by minimizing the influence of background ions and natural organic matter. Therefore, the results should be interpreted as a controlled mechanistic assessment of multi-metal adsorption behavior rather than a direct representation of environmental conditions.

## Materials and methods

### Materials and reagents

To examine the interaction between polymeric particles and heavy metals, pristine polypropylene (PP) and polyvinyl chloride (PVC) obtained from industrial suppliers were selected as the sorbent materials. Table S1 presents a summary of the size distribution measurements performed on the MasterSizer 2000 device. The mean particle size (d_50_) of PP was determined to be 116 μm (8.7–724 μm), while that of PVC was 157 μm (63–363 μm).

Co(NO_3_)_2_·6H_2_O (Honeywell/Fluka, ACS reagent, ≥ %98), Ni(NO_3_)_2_·6H_2_O (ZAG Chemistry, %99), Ba(NO_3_)_2_ (Alfa Aesar, %99) and Mn(NO_3_)_2_·4H_2_O (Sigma-Aldrich, ≥ %97) were used to prepare metal solutions. 0.1 M HCl (%37, Merck) and 0.1 M NaOH (Tekkim) were used to adjust the pH of metal solutions. A bench type pH meter (pH 720 WTW Series, inoLab) was employed for pH measurement. A glass microfiber filter (47 mm, 0.45 µm pore size, Whatman, China) was utilized for sample filtration in the experiments.

### Material characterization

A comprehensive analysis was conducted to determine the characteristics of pristine or metal-loaded MPs. The evaluation of specific surface area was conducted using the Quantachrome TouchWin v1.2 device. This was accomplished through two approaches: first, by determining nitrogen gas adsorption–desorption isotherms utilizing the BET method; second, by assessing the actual volume and density of the materials with the Quantachrome Ultra PYC 1200e gas pycnometer. The specific surface area measurements yielded a value of 16.671 m^2^/g for PP, whereas the surface area for PVC could not be calculated (< 1 m^2^/g). The inability of PVC particles to effectively absorb nitrogen gas suggests the absence of accessible pore structures, which was further supported by volume and density measurements obtained from gas pycnometry. The nitrogen adsorption method is applied for materials exhibiting smaller pore sizes (< 300 nm), whereas the mercury porosimetry method is appropriate for larger pores (< 200 µm) (Tseng et al., 2022).

The point of zero charge (pzc) was determined using a pH drift (salt addition) method, which is widely applied in adsorption studies involving polymeric and particulate materials. Briefly, the pzc was established by calculating the difference between the initial and the final pH after the addition of approximately 10 mg of MPs to a 20 mL solution of 0.1 M NaCl, with pH value ranging from 2 to 11, under stirring conditions. The pH_pzc_ was determined to be 6.41 for PP and 6.39 for PVC. Although this approach provides an operational estimation of surface charge neutrality rather than a full electrokinetic characterization, it is commonly used due to its simplicity, reproducibility, and suitability for heterogeneous particulate systems. Based on the obtained pHpzc values, experiments were conducted at pH 7 to ensure predominantly negatively charged MPs surfaces under the experimental conditions, favoring electrostatic interactions with metal cations.

The visual examination of MPs was conducted using the optical component of a scanning electron microscope, whereas their chemical structure was evaluated through FTIR-ATR spectroscopy (Shimadzu IR Tracer-100 model). The shape and elemental composition of the MPs were analyzed using SEM/EDX techniques (Godoy et al., 2019; Veerasingam et al., 2021) after rinsing with deionized water. In the analysis, pristine or metal-loaded MPs were affixed to a conductive and sticky carbon tape positioned on an aluminum sample holder. All samples were coated with 5 nm gold to enhance absorption. Morphological and microstructural analyses were conducted utilizing secondary electron imaging at 20 kV, with a working distance of 7–9 mm, alongside energy dispersive X-ray spectroscopy (EDX, Oxford Instruments, INCA) techniques in a scanning electron microscope (SEM, Zeiss, SUPRA 50 V P) to identify heavy metals on the MP surface. In EDX analyses, 21 particles from each MP in the experimental group were randomly chosen, and their surfaces were scanned for 20 s for selected elements (C, Mn, Ba, Co, Ni) automatically generated by the software. On the other hand, the functional groups in PP and PVC were analyzed at a frequency of 400–4000 cm^−1^ with a spectral resolution of 4 cm^−1^. The spectrum was subsequently compared with the polymer library, and a visual inspection was performed to examine the characteristic bands in the reference spectra. Only data points with a matching degree, 70% or above, were acceptable.

### Adsorption studies

The kinetics of metal interactions with MPs at a fixed initial concentration of 1 mg/L were conducted in 42 mL dark glass bottles, establishing a solid/liquid ratio of 1 g of MPs per 100 mL of solution. Experiments were performed in Milli-Q deionized water at pH 7 and 25 °C using a WTW TS606/2-I thermo incubator and a 150 rpm shaker (WTW TR-1 Heidolph UNIMAX 2010). Samples were collected at predetermined time intervals (0.25, 1, 3, 6, 12, 24, 48 h) to determine the equilibrium time (Zou et al., 2020). Adsorption experiments were conducted across a concentration range of 0.5–75 mg/L. This range was designed to provide sensitivity across low-level exposures while ensuring a sufficiently broad gradient for adsorption isotherm analysis, surface loading detection, and inter-study comparability. These concentrations are not intended to represent typical background levels in pristine natural waters; rather, they constitute a controlled screening range applicable to contaminated or effluent-impacted scenarios and to facilitate characterization of polymer- and metal-dependent adsorption behavior. Batch experiments were conducted using Milli-Q water to establish a controlled baseline system and minimize interference from dissolved constituents that may affect adsorption behavior. The selected MP dosage and metal concentration range are consistent with those commonly reported in batch adsorption studies (Fan et al., 2021a, 2021b; Gao et al., 2019; Li et al., 2020; Tenea et al., 2024; Wang et al., 2017), particularly for mechanistic investigations and multi-metal systems, where elevated concentrations (typically 10–100 mg/L) are used to ensure quantifiable adsorption capacity and enable reliable kinetic and isotherm modeling. Samples collected during the experiments underwent filtration followed by analysis using the ICP-OES Spectrophotometer (VARIAN 720 ES instrument). The calibration parameters can be found in the Supplementary Material (Table S2). The analytical performance of ICP-OES was evaluated in terms of the limit of detection (LOD) and measurement uncertainty. LOD values were calculated based on three times the standard deviation of procedural blanks divided by the slope of the calibration curve, yielding 0.0031 mg/L for Co, 0.0003 mg/L for Mn, 0.004 mg/L for Ni, and 0.0016 mg/L for Ba. Relative measurement uncertainties were determined as 1.57% for Co, 1.78% for Mn, 1.43% for Ni, and 1.66% for Ba. These uncertainty levels are low relative to the observed concentration changes, indicating that analytical variability does not significantly affect the interpretation of adsorption behavior. Each experiment was conducted in duplicate, and the reported values represent mean values. Standard deviations were calculated and are presented as error bars in figures, and are also provided in the Supplementary Material for all kinetic and isotherm datasets. The maximum amount of heavy metal adsorbed at equilibrium (q_e_) for PP and PVC was calculated by the formula given below (Özüdoğru et al., 2022):

$$ {\mathrm{Adsorption}}\,{\mathrm{capacity}},{\mathrm{q}}_{{\mathrm{e}}} \left( {{\mathrm{mg}}/{\mathrm{g}}} \right) = \frac{{\left( {{\mathrm{C}}_{0}  - {\mathrm{C}}_{{\mathrm{e}}} } \right) \cdot {\mathrm{V}}}}{{\mathrm{W}}}  $$

where C_0_ and C_e_ are the initial and equilibrium heavy metal concentration (mg/L), V is the total volume of adsorbate solution (L) and W is the amount of adsorbent (PP or PVC) (g).

All kinetic and equilibrium adsorption models were fitted using non-linear regression analysis applied directly to the original experimental datasets (q_t_-t for kinetics and q_e_-C_e_ for isotherms) without any linearization or data transformation. This approach was adopted to avoid distortion of experimental error structure, unequal weighting of data points, and potential bias in parameter estimation commonly associated with linearized fitting procedures. All model fitting and statistical evaluations were performed using the Python programming language, and the analyses were executed within the PyCharm integrated development environment (PyCharm 2025.2.0.1).

Non-linear kinetic modeling was performed using the pseudo-first-order (PFO), pseudo-second-order (PSO), and Elovich models, while equilibrium data were fitted using the Langmuir, Freundlich, and Temkin isotherm models in their non-linear forms. Parameter estimation for adsorption isotherms was carried out using the curve_fit() function in the SciPy library, which applies the Levenberg–Marquardt algorithm for non-linear least-squares optimization. For kinetic models, a derivative-free Nelder–Mead simplex algorithm was employed to facilitate robust parameter estimation for the kinetic datasets. All model parameters were iteratively optimized until convergence was achieved based on predefined tolerance criteria. Model performance was evaluated using multiple statistical error functions, including the coefficient of determination (R^2^), root mean square error (RMSE), hybrid fractional error (HYBRID), Marquardt’s percent standard deviation (MPSD), and average relative error (ARE). The use of multiple error functions minimizes model selection bias and provides a more robust evaluation of fitting performance compared to reliance on a single statistical indicator (Shahmohammadi-Kalalagh & Babazadeh, 2014). In addition, R^2^ alone may not provide a reliable basis for evaluating non-linear adsorption models, and therefore model selection in the present study was based on the combined evaluation of R^2^ together with RMSE, HYBRID, MPSD, and ARE (Spiess & Neumeyer, 2010). The mathematical formulations of the applied kinetic and isotherm models are provided in Tang et al. (2020) and López-Luna et al. (2019), and are also explicitly presented in the Supplementary Material (Table S3).

## Results and discussion

### Adsorption kinetics

**Fig. 1:**
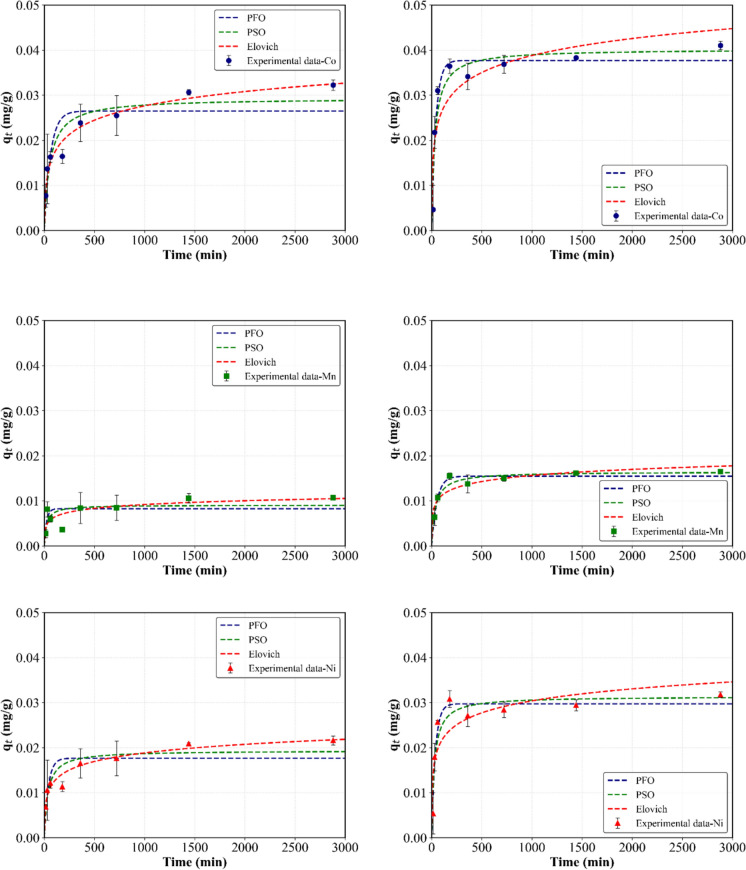
Pseudo-first order, pseudo-second order and Elovich kinetic models on PVC (left panel) and PP (right panel)

The kinetics of heavy metal adsorption onto MPs involve an examination of the rate of adsorption onto surfaces and elucidating the mechanisms that govern this interaction. Kinetic experiments were conducted at an initial metal concentration of 1 mg/L, pH 7, 25 °C, and an MP dosage of 10 g/L under constant agitation. The results of kinetic study exhibited rapid adsorption during the initial stage (first few hours), followed by a deceleration phase between this initial period and 48 h, ultimately approaching equilibrium (Fig. S1). While the exact transition point of this initial phase may vary slightly depending on the metal–polymer system, the overall trend indicates a fast initial uptake followed by a slower approach to equilibrium. This two-stage adsorption behavior reflects the progressive occupation of available surface sites on PP and PVC, where an initially high driving force due to concentration gradients is gradually reduced as the system approaches equilibrium (Wang et al., 2022). Accordingly, the time was set at 48 h to ensure that the process could reach complete equilibrium. In the literature, the equilibrium times observed in single and multi-metal adsorption studies exhibit considerable variation, which is influenced by a number of factors, including the nature of the adsorbent, the characteristics of the adsorbate, and the prevailing environmental conditions. The time values reported across studies vary considerably, with different adsorption durations documented, including 9 days, 720 min and 48 h (Fan et al., 2021a; Wang et al., 2022). As presented in Fig. 1, the adsorption profiles indicate metal-dependent uptake behavior among the investigated ions, suggesting variations in apparent adsorption tendencies under identical experimental conditions. The disparity in affinity between PP and PVC is attributed to the physicochemical characteristics, including material composition, surface structure, surface area and functional groups (Wang et al., 2022). Due to matrix-related signal fluctuations and element-specific variability observed for Ba (with relative deviation ranging from approximately 1–8% during calibration and measurement), reliable kinetic modeling could not be achieved without introducing substantial uncertainty into model parameters (Fig. S1). Therefore, Ba was excluded from kinetic model evaluation, while its results was still considered in subsequent discussions.

Non-linear kinetic modeling was conducted using the pseudo-first-order (PFO), pseudo-second-order (PSO), and Elovich models, and the derived parameters are presented in Table S4. Model selection was based on a combined evaluation of R^2^ and multiple error functions (RMSE, HYBRID, MPSD, and ARE), which consistently supported the identified best-fitting models. The kinetic rate constants obtained from PFO, PSO, and Elovich models were found to be relatively close for all investigated metals, ranging between 0.016 and 0.044 min^−1^. This narrow range suggests limited differentiation in the apparent adsorption rates among Mn, Ni, and Co under the studied conditions. Accordingly, kinetic modeling results are interpreted as descriptive representations of adsorption rate behavior rather than indicators of distinctly different rate-controlling mechanisms.

For PVC, the Elovich model provided the best overall fit (higher R^2^ and lower error values across statistical functions), indicating consistent behavior with the low accessible surface area and heterogeneous surface characteristics of PVC, where adsorption is likely governed by a distribution of energetically non-equivalent sites rather than uniform surface coverage (Shao et al., 2023). In contrast, for PP, the PFO model showed the most consistent performance for Co, Ni, and Mn, with systematically higher R^2^ values and lower error metrics (i.e., RMSE, HYBRID, MPSD, ARE), suggesting that this model provides a better empirical representation of the adsorption rate data under the studied conditions. Although PSO also yielded acceptable fits for some datasets, its overall error functions were less favorable than those of PFO. It should be emphasized that kinetic models such as PFO, PSO, and Elovich are primarily empirical and are used here to describe adsorption rate behavior rather than to directly identify a unique rate-limiting mechanism. Therefore, mechanistic interpretation should be made cautiously, as these models alone cannot explicitly distinguish between film diffusion, intraparticle diffusion, or surface reaction-controlled processes. Similar variability in kinetic model suitability has been reported in the literature, where different MP types and metal species were found to follow different kinetic descriptions. These findings support that adsorption kinetics on MPs are system-specific and depend on both polymer characteristics and metal properties rather than conforming to a single universal kinetic model (Cao et al., 2021; Liu et al., 2022; Shao et al., 2023). Despite the success of models in describing kinetic processes involving heavy metals, it should be noted that they are predominantly developed for single-ion systems. The incorporation of multi-ion effects into mechanistic kinetic models would improve their applicability in complex systems.

### Adsorption isotherms

Figure S2 depicts the adsorption isotherms of heavy metals on PVC and PP under equilibrium conditions at a contact time of 48 h, MP concentration of 10 g/L, and initial metal concentrations ranging from 0.5 to 75 mg/L at pH 7. The isotherms indicate that each heavy metal exhibits a distinct adsorption capacity, reflecting differences in metal-surface affinity under multi-metal conditions. As shown in Fig. S2, q_e_ increased rapidly with increasing metal concentration and gradually approached a plateau at higher concentrations, indicating progressive occupation and eventual saturation of available adsorption sites on the MP surfaces. Given the limited number of experimental replicates (n = 2), the observed differences should be interpreted as descriptive trends rather than statistically significant distinctions.

**Fig. 2:**
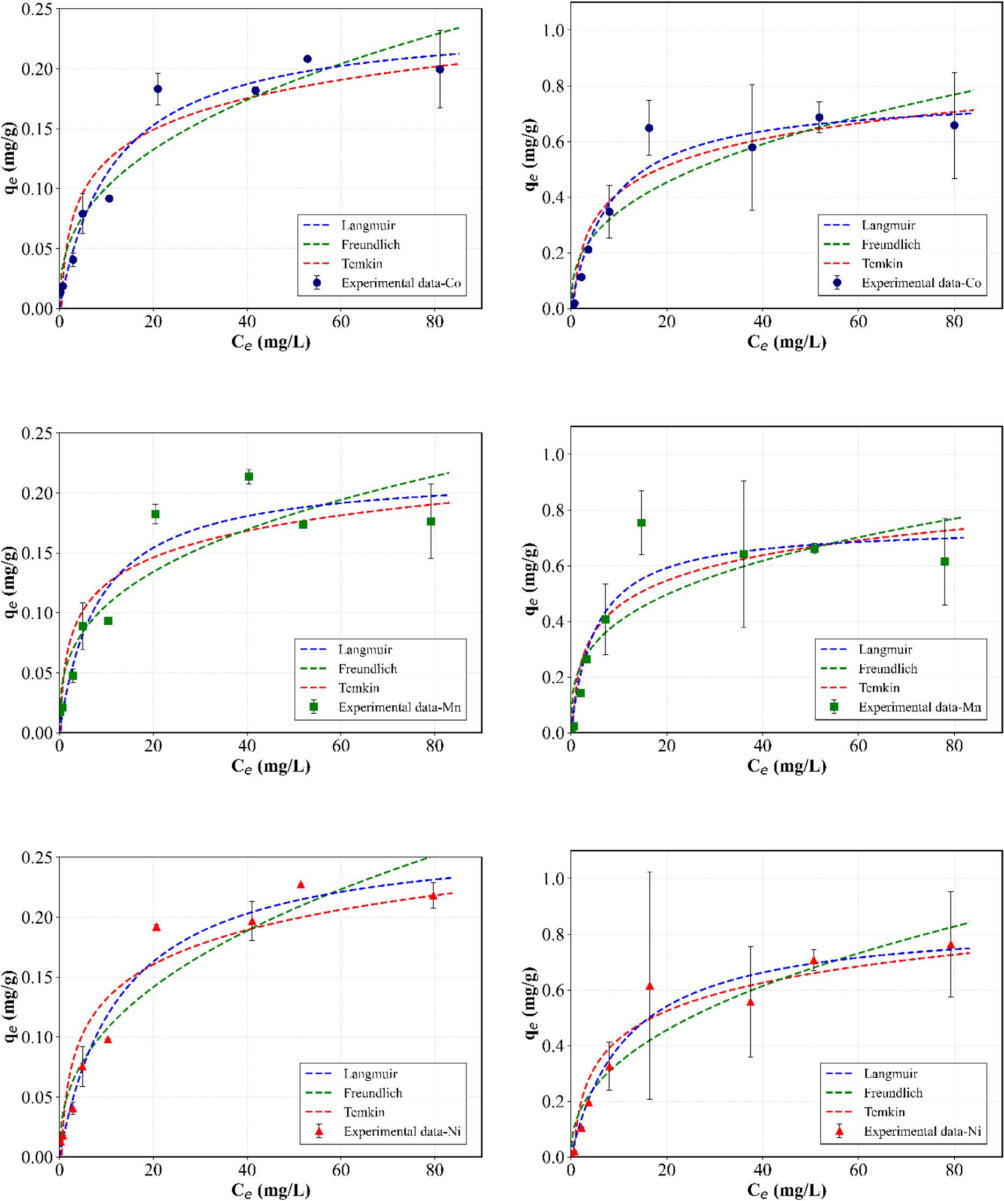
Langmuir, Freundlich and Temkin isotherm models on PVC (left panel) and PP (right panel)

Three commonly used equilibrium isotherm models (Langmuir, Freundlich and Temkin) were employed to analyze the adsorption characteristics of heavy metals more effectively, as they represent different assumptions regarding surface homogeneity and adsorbate–adsorbent interactions. The coefficients for these models, as shown in Table S5, were derived from the graphs presented in Fig. 2. The superior performance of the Langmuir model was consistently supported across all applied statistical criteria (R^2^, RMSE, HYBRID, MPSD, and ARE), rather than relying on R^2^ alone. The Langmuir K_L_ values obtained for PVC (0.0766–0.1210 L/mg) and PP (0.0865–0.1946 L/mg) indicate generally comparable adsorption affinities among Co, Ni, and Mn, with Mn exhibiting relatively higher values, particularly for PP. In contrast, Co and Ni show closer K_L_ values, suggesting similar affinity under the investigated multi-metal conditions. Overall, these results indicate that while a general adsorption trend is observed, metal-specific deviations are present, particularly for Mn in PP systems.

An evaluation of Table S5 indicates that the Langmuir isotherm consistently provided the best overall fit across all investigated metals, as supported by multiple statistical criteria (R^2^, RMSE, HYBRID, MPSD, and ARE). Notably, PP exhibited numerically higher Langmuir model-derived capacities (q_m_) than PVC for all investigated metals, suggesting a tendency toward higher equilibrium uptake, despite differences in adsorption kinetics and surface interaction mechanisms. It should be noted that although these isotherm models are widely used for equilibrium interpretation, they are fundamentally developed for single-solute adsorption systems. Since the present experiments were conducted under a multi-metal system, the derived parameters (including q_m_) should be interpreted as apparent adsorption capacities reflecting coexisting ion uptake behavior rather than intrinsic single-metal affinities under idealized systems. These parameters represent the integrated response of Mn, Ni, Co, and Ba in the same solution, potentially reflecting competition for available adsorption sites on PP and PVC surfaces. In comparison with previously reported studies, the adsorption capacities obtained in this study fall within the lower range of values typically reported for MPs. This difference is expected, as single-metal systems do not involve coexisting ions competing for adsorption sites, allowing higher apparent sorption capacities. Under multi-metal conditions, the presence of coexisting ions leads to competitive interactions that reduce the effective adsorption of individual metals. Although data exist for some metals such as Ni and Co, data for Mn and particularly Ba remain limited, and studies addressing their behavior under multi-metal conditions are still scarce. A detailed comparison with literature values is provided in the Supplementary Material (Table S6). Based on the combined evaluation of equilibrium uptake and isotherm-derived parameters, a general adsorption tendency of Co, Ni, and Mn relative to Ba (e.g., Co ≥ Ni ≥ Mn > Ba) was observed; however, differences among Co, Ni, and Mn remained minor and dependent on the specific descriptor used.

The Langmuir isotherm assumes adsorption onto a finite number of energetically equivalent sites, resulting in monolayer coverage without lateral interactions between adsorbed species. Within this framework, the higher specific surface area of PP compared to PVC likely contributes to the higher apparent q_m_ values observed for PP under equilibrium conditions, as increased surface area enhances the accessibility of adsorbate molecules to available active sites (Deshpande et al., 2011). When considered together, the kinetic and isotherm results indicate that adsorption behavior differs between PVC and PP in terms of how equilibrium is reached and described statistically. While the Langmuir isotherm suggests a well-defined equilibrium uptake for both MPs, the kinetic modeling reveals polymer-specific differences in the dominant empirical description of adsorption rates. This divergence indicates that equilibrium capacity and adsorption kinetics respond differently to metal-surface interactions, emphasizing that adsorption behavior on MPs cannot be fully described by a single model or parameter. Although the Langmuir model assumes energetically equivalent adsorption sites at equilibrium, its superior statistical performance does not necessarily exclude the presence of surface heterogeneity during the adsorption process, which is more effectively reflected by kinetic models such as Elovich.

### Characterization and mechanism of MP-metal interaction

#### SEM–EDX analysis

**Fig. 3:**
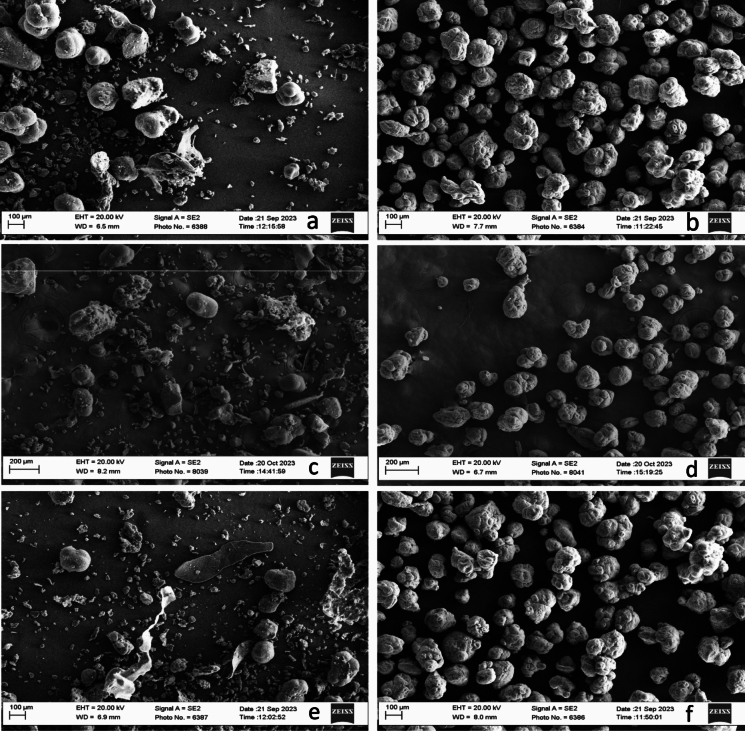
SEM images of PP and PVC microplastics before and after metal adsorption. **a** pristine PP (scale bar: 100 μm; magnification: 285X); **b** pristine PVC (100 μm; 198X); **c** PP exposed to 100 mg/L metal solution (200 μm; 150X); **d** PVC exposed to 100 mg/L metal solution (200 μm; 125X); **e** PP exposed to 0.5 mg/L metal solution (100 μm; 320X); **f** PVC exposed to 0.5 mg/L metal solution (100 μm; 250X)

The high-resolution SEM images combined with EDX analysis provide detailed insights into the surface morphology and elemental composition of metal-loaded particles (Gniadek & Dąbrowska, 2019). Accordingly, Fig. 3 compares the morphological characteristics of pristine and metal-loaded PP and PVC particles.

Pristine PP particles exhibited a more irregular morphology with relatively smoother surfaces and a broad size distribution, whereas PVC particles showed more uniform and regular shapes, often appearing spherical. Following metal exposure, PP particles developed increased surface roughness (Fig. 3c and e), which is known to enhance adsorption by increasing the number of accessible surface sites. Previous studies have reported that smaller PP particles exhibit higher adsorption capacities for heavy metals such as Pb, Cu, and Zn compared to PVC, primarily due to size-dependent surface area effects (Barus et al., 2023; Liu et al., 2022). Consistent with these findings, PP particles in the present study generally indicated a higher metal uptake than PVC, which can be attributed to their smaller particle size and heterogeneous surface morphology (Fig. 3c–f).

**Fig. 4:**
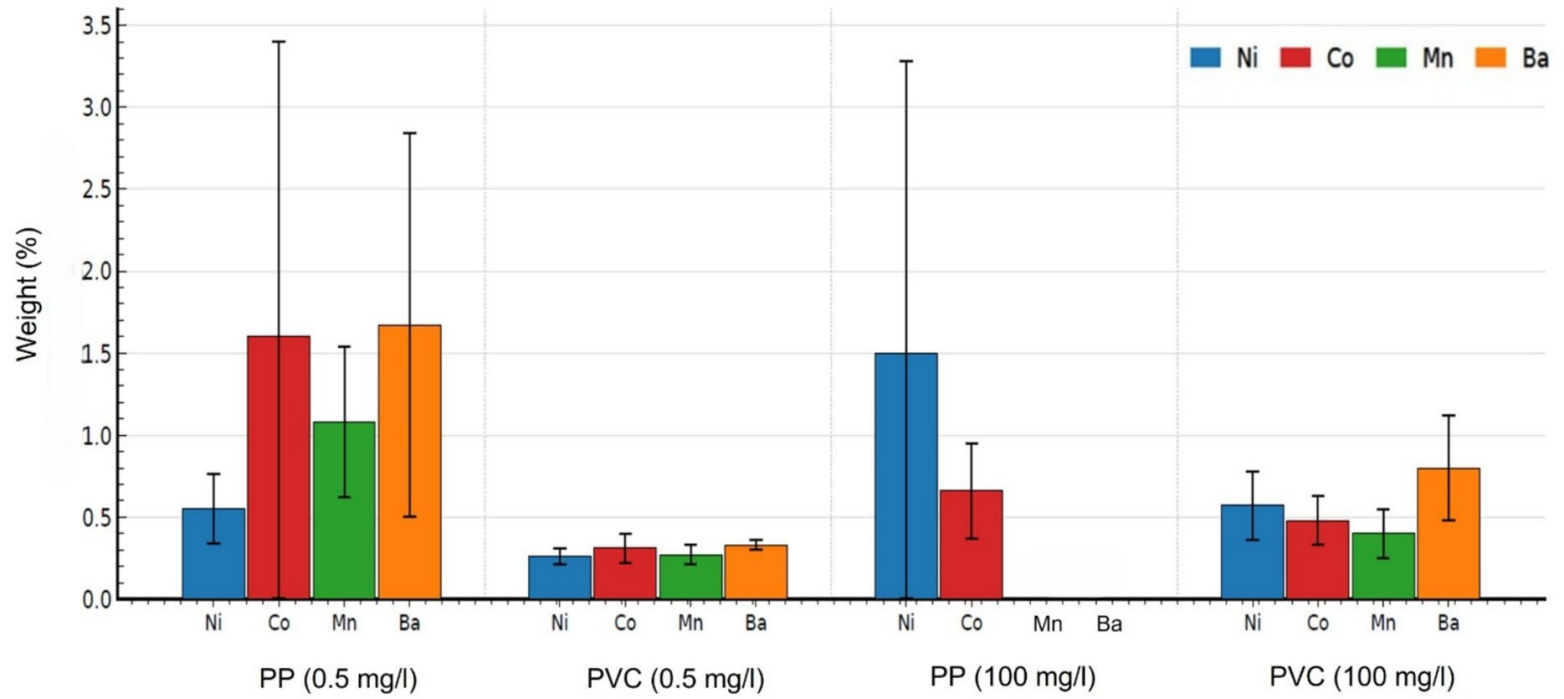
Mean heavy metal adsorption on PP and PVC with standard deviation (± SD) based on particle-level EDX measurements. Error bars represent variability among individually analyzed particles (n = 6–13 per condition)

In the EDX analysis, 21 particles were randomly examined for each condition. Among them, 10 particles in the PVC-0.5 mg/L group, 13 in the PVC-100 mg/L group, and 6 particles in both PP exposure groups exhibited detectable metal adsorption (Figs. S3–S6). As shown in Fig. 4, metal loadings on PP particles showed greater variability. In some cases, the standard deviation exceeds the mean value, particularly for PP at 0.5 mg/L Co (mean = 1.60 wt %, SD = 1.80 wt %) and PP at 100 mg/L Ni (mean = 1.50 wt %, SD = 1.78 wt %). This variability reflects the inherent heterogeneity of PP microplastics and the particle-based nature of SEM–EDX analysis. Given the limited number of analyzed particles (n = 6–13 per condition), and the proximity of several measurements to the EDX detection limit, only a subset exhibited detectable metal signals. The remaining particles were at or near the detection limit which further amplifies variability in semi-quantitative EDX measurements, particularly at low surface loadings. As a result, the data distribution is highly skewed, with a few particles showing elevated metal loadings and others showing minimal or no detectable adsorption, leading to relatively large standard deviations. This behavior is consistent with the irregular morphology, broad particle size distribution (8.71–724 μm), and higher surface heterogeneity of PP compared to PVC. In contrast, PVC showed a narrower distribution of metal contents with lower standard deviations, consistent with its more uniform morphology. Although fewer PP particles exhibited detectable metal signals, those particles tended to show higher metal loadings on a per-particle basis, indicating a greater adsorption capacity at the particle scale. This observation aligns with the isotherm results, which indicated higher equilibrium uptake for PP. While PVC exhibited stronger metal-surface interactions due to its polar C–Cl groups, PP exhibits higher overall metal accumulation, likely due to its larger effective surface area and heterogeneous morphology (Chen et al., 2023). The observed variability therefore reflects particle-to-particle differences in adsorption behavior rather than uniform surface coverage, and is consistent with the heterogeneous adsorption mechanisms inferred from kinetic modeling (PFO for PP vs. Elovich for PVC). It should be noted that SEM–EDX provides semi-quantitative surface information and cannot distinguish between strongly bound and potentially loosely associated surface species. Therefore, the detected metal signals should be interpreted as indicative of surface-associated metals rather than definitive evidence of specific adsorption mechanisms or binding configurations.

#### FTIR analysis

To improve spectral comparability, FTIR spectra of pristine and metal-exposed samples are presented in the same plots (Figs. S7 and S8), together with major band assignments (e.g., C–H stretching at ~ 2950–2850 cm^−1^ and bending vibrations at ~ 1450–1375 cm^−1^). Spectroscopic analysis of pristine PP and PVC showed matching ratios of 75% and 87%, respectively, with their reference spectra. Following metal adsorption, a decrease in spectral similarity was observed for both polymers, with a more pronounced reduction for PP. The matching ratio of PP decreased to 70% at 0.5 mg/L and further to 67% at 100 mg/L metal exposure, whereas PVC exhibited only minor changes (85% and 86% at 0.5 and 100 mg/L, respectively). No significant peak shifts were observed after metal exposure, which is consistent with the expected non-specific and predominantly electrostatic nature of metal-MP interactions. Therefore, the observed variations are interpreted in terms of relative intensity changes and spectral similarity rather than band displacement. These changes in spectral similarity may indicate surface-level modifications, which may reflect a combination of factors including metal adsorption, partial leaching of surface additives during aqueous exposure, and morphological changes affecting ATR contact efficiency. The pronounced reduction in matching ratio for PP compared to PVC is consistent with its higher surface reactivity and adsorption capacity observed through SEM–EDX and ICP-OES measurements. However, given the predominantly electrostatic and physical nature of metal-MP interactions (as indicated by kinetic and isotherm modeling), and the absence of abundant oxygen-containing functional groups on pristine PP and PVC, FTIR-ATR does not provide definitive evidence for specific metal–ligand coordination. Therefore, FTIR-ATR is inherently limited in resolving adsorption mechanisms involving non-specific interactions and should be interpreted as a complementary indicator of surface modification rather than a definitive mechanistic probe.

Minor variations in band intensity, particularly in regions associated with O–H stretching (~ 3350 cm^−1^), may reflect changes in surface-associated functional groups or adsorbed species (Rao, 2021). However, these changes are not sufficient to confirm specific binding mechanisms. Similarly, slight variations in C–H stretching vibrations suggest localized changes in the polymer environment rather than structural transformation. In contrast, PVC exhibited relatively stable FTIR spectra, consistent with limited structural perturbation despite the presence of polar C–Cl groups that may facilitate electrostatic interactions (Wang et al., 2023). The limited changes in peak position and intensity indicate that these interactions do not significantly alter the overall molecular structure of PVC. Overall, the FTIR results support the conclusion that PP undergoes more pronounced surface-level alterations upon metal adsorption compared to PVC, in agreement with its higher surface reactivity and adsorption capacity (Chen et al., 2023; Gao et al., 2019). Nevertheless, the observed spectral variations should be interpreted as indirect evidence of surface modification rather than definitive identification of adsorption mechanisms.

#### Other factors influencing multi-metal adsorption

The adsorption behavior of MP types may be influenced by multiple factors, including polymer structure, crystallinity, surface chemistry, and environmental conditions. PP generally exhibits a higher degree of crystallinity (30–70%), which provides enhanced mechanical stability but may limit the accessibility of internal adsorption sites due to tightly packed crystalline domains (Moura et al., 2023). In contrast, PVC typically shows lower crystallinity (10–15%) and a more amorphous structure resulting from the presence of chlorine atoms, which disrupt polymer chain packing (Ramesh et al., 2010). Although amorphous regions are often associated with increased surface reactivity, adsorption performance is not solely determined by crystallinity. In the present study, PP exhibited higher overall metal uptake, which can be attributed to its heterogeneous surface morphology, broader particle size distribution, and larger effective surface area, as supported by SEM–EDX and isotherm results. Conversely, PVC appeared to indicate stronger metal-surface interactions due to its polar C–Cl groups, facilitating electrostatic attraction and possible complexation, but with comparatively lower total adsorption capacity. These observations suggest that adsorption capacity and interaction strength do not necessarily scale together and may respond differently to polymer-specific properties under the investigated conditions.

In addition to polymer characteristics, metal-specific properties may also influence adsorption behavior under multi-metal conditions. Under the experimental conditions (pH 7, 25 °C), aqueous speciation may also influence the adsorption behavior of the investigated metals. Speciation calculations (Visual Minteq 4.0) performed for the multi-metal nitrate system indicate that Mn, Ni, Co, and Ba are predominantly present as free divalent cations (> 99%), with only minor contributions from first hydrolysis species, such as CoOH^+^ and NiOH^+^, while MnOH^+^ and BaOH^+^ remain negligible. Nitrate complexation is also minimal under these conditions. Given that the solution pH is slightly above the pHpzc of the MPs (~ 6.4), the MP surfaces are expected to be negatively charged, favoring electrostatic interactions with cationic species. Therefore, adsorption is expected to be predominantly governed by interactions among hydrated divalent metal ions, while differences in adsorption behavior are likely associated with variations in hydrated ionic radius, hydration energy, and hydrolysis tendency rather than major differences in aqueous speciation. The hydrated ionic radius affects the ability of metal ions to approach and access adsorption sites, with larger hydrated ions experiencing greater steric hindrance. Accordingly, Ba, with the largest hydrated radius (~ 5 Å), exhibited lower adsorption efficiency compared to Mn, Ni, and Co, which possess smaller hydrated radii (Chen et al., 2024; Musumba et al., 2020). Similarly, smaller ionic radii may facilitate closer interaction with surface binding sites, further enhancing adsorption (Meng et al., 2021).

Electronegativity may further contribute to adsorption behavior by influencing the strength of metal-surface interactions. Transition metals such as Ni, Co, and Mn, with higher electronegativity values, may exhibit a greater tendency to interact with polar functional groups, particularly on PVC surfaces, whereas Ba may exhibit weaker interactions due to its lower electronegativity (Fan et al., 2021b; Liu et al., 2021a). Under neutral pH conditions, partial deprotonation of surface functional groups may further promote interactions with transition metals.

Taken together, the SEM–EDX observations, adsorption isotherms, and multi-metal adsorption analyses suggest that metal uptake by PP and PVC microplastics is influenced by the interplay between polymer-specific surface characteristics and metal-specific physicochemical properties. PP exhibited higher overall adsorption capacity, which may be linked to its heterogeneous morphology, broader particle size distribution, and larger effective surface area. In contrast, PVC showed indications of stronger metal-surface interaction strength due to the presence of polar C–Cl functional groups, despite its comparatively lower total uptake. The observed adsorption behavior among Mn, Ni, Co, and Ba in the presence of multiple metal ions appears to be modulated by hydrated ionic radius, ionic radius, and electronegativity, with the transition metals generally exhibiting higher adsorption than Ba. However, given the limited number of experimental replicates and the relatively small differences among Co, Ni, and Mn, these trends should be interpreted as indicative rather than statistically definitive. It should be emphasized that the proposed interpretations are based on indirect evidence derived from adsorption behavior, surface characterization, and known physicochemical properties, rather than direct spectroscopic identification of binding mechanisms at the molecular level.

Although the adsorption capacities of PP and PVC microplastics are relatively lower compared to natural sorbents such as clay minerals, organic matter, and sediments, which possess higher surface area and a greater density of reactive functional groups, the environmental relevance of MPs as metal carriers may be better understood within a broader system perspective. In aquatic environments, MPs are highly persistent, widely distributed, and continuously generated through fragmentation processes, leading to their coexistence with natural particulate matter in multi-component systems. Under such conditions, MPs may act as supplementary sorbent phases, contributing to the redistribution and transport of trace metals, particularly in environments subject to significant anthropogenic inputs. Therefore, although MPs do not represent the dominant sorption phase, their role as persistent and mobile carriers may still be environmentally relevant in terms of contaminant fate and transport (Li et al., 2018; Rochman et al., 2019). Collectively, these factors are consistent with the observed adsorption behavior, where Co, Ni, and Mn generally exhibit higher uptake compared to Ba, although differences among transition metals remain relatively small under the studied conditions.

## Conclusion

This study investigated the adsorption behavior of Mn, Ba, Ni, and Co ions on pristine PP and PVC microplastics in a multi-metal system. The results indicate that Ba consistently exhibited lower adsorption compared to Mn, Ni, and Co, while only minor differences were observed among the transition metals under the investigated conditions. These findings provide new insight into multi-metal adsorption on MPs, addressing an important gap in the current literature that predominantly focuses on single-metal systems.

Kinetic modeling revealed polymer-dependent adsorption behavior, with the pseudo-first-order model providing the most consistent description for PP, while the Elovich model better captured the adsorption kinetics of PVC, reflecting differences in surface heterogeneity and interaction behavior. In contrast, equilibrium adsorption for both MPs was well described by the Langmuir isotherm. However, this should be interpreted as apparent Langmuir-type behavior under multi-metal conditions rather than evidence of monolayer adsorption on heterogeneous MP surfaces. SEM–EDX analysis showed a more extensive surface distribution of adsorbed metals on PP compared to PVC, attributable to its higher surface area-to-mass ratio and heterogeneous morphology. FTIR results further supported these observations, with PP exhibiting changes in C–H stretching regions after metal adsorption, whereas PVC showed variations mainly in C–H bending and C–C stretching regions, particularly in the presence of Ni, reflecting polymer-specific interaction characteristics.

Although the experiments were conducted under controlled laboratory conditions, which may not fully represent environmental complexity, the results highlight the importance of polymer structure and metal physicochemical properties in governing adsorption behavior in multi-metal systems. Moreover, the limited number of experimental replicates and the absence of single-metal adsorption data restrict the statistical strength and direct quantification of competitive effects. Therefore, the observed polymer- and metal-dependent differences should be interpreted as indicative trends requiring confirmation through future studies with larger replication and formal statistical evaluation. Future studies should incorporate advanced surface-sensitive techniques (e.g., XPS) for bonding analysis, along with environmentally relevant factors such as background ions, natural organic matter, aging processes, temperature fluctuations, and biofilm formation to better assess the long-term environmental behavior of MPs. Overall, while MPs do not represent dominant sorbent phases compared to natural materials, their persistence and mobility suggest that they may contribute as supplementary carrier phases influencing the redistribution and transport of trace metals under multi-component environmental conditions.

## Supplementary Information

Below is the link to the electronic supplementary material.

Supplementary file1 (DOCX 4131 KB)

## Data Availability

No datasets were generated or analysed during the current study.
