# Supplementary material for: Multi-metal adsorption behavior of PP and PVC microplastics: polymer-dependent interactions and mechanisms
Source: Environ Geochem Health. 2026 Jun 30;48(10):426. doi: 10.1007/s10653-026-03316-3 (PMC13319911; doi:10.1007/s10653-026-03316-3)
Supplement: Supplementary file 1 — Supplementary file1 (DOCX 4131 KB) [file 10653_2026_3316_MOESM1_ESM.docx]

SUPPLEMENTARY MATERIAL

Multi-metal adsorption behavior of PP and PVC microplastics: polymer-dependent interactions and mechanisms

İlknur Demirtaş^1^, Parisa Akbari Dana^1^, Zehra Yiğit Avdan^1,2^, Kadir Gedik^1,2*^

^1^Eskişehir Technical University, Department of Environmental Engineering, 26555 Eskişehir, Türkiye

^2^Eskişehir Technical University, Environmental Research Center (ÇEVMER), 26555 Eskişehir, Türkiye

* Corresponding author.

E-mail: kgedik@eskisehir.edu.tr

Table S1. Particle size distribution for PP and PVC.

| Polymer | Min-Max (μm) | d_10%_ (μm) | d_50%_ (μm) | d_90%_ (μm) |
| --- | --- | --- | --- | --- |
| PP | 8.71-724 | 26.6 | 116 | 378 |
| PVC | 63.0-363 | 105 | 157 | 234 |

Table S2. The calibration data for Ba, Co, Mn, and Ni in the ICP-OES spectrophotometer (Varian 720 ES)

| Ba 493.408 Calibration (mg/L) |  |  |  |  |  | |  |
| --- | --- | --- | --- | --- | --- | --- | --- |
| Standard | Flaqs | Int (c/s) | Std Conc | Calc Conc | Error | | %Error |
| Standard 1 | --- | 143458.7 | 0.100000 | 0.092285 | -0.008 | | -7.715 |
| Standard 2 | --- | 426774.8 | 0.300000 | 0.308524 | 0.009 | | 2.841 |
| Standard 3 | --- | 823895.4 | 0.600000 | 0.611624 | 0.012 | | 1.837 |
| Standard 4 | --- | 1350804.0 | 1.00000 | 1.01386 | 0.014 | | 1.386 |
| Standard 5 | --- | 2628123.0 | 2.00000 | 1.98869 | -0.01 1 | | -0.565 |
| Correlation Coefficient | 0.999855 |  |  |  |  | |  |
| Status | Calibrated |  |  |  |  | |  |
| Curve Type | Linear |  |  |  |  | |  |
| Co 228.615 Calibration (mg/L) |  |  |  |  |  | |  |
| Standard | Flaqs | Int (c/s) | Std Conc | Calc Conc | | Error | %Error |
| Standard 1 | --- | 384.9 | 0.100000 | 0.089962 | -0.010 | | -10.038 |
| Standard 2 | --- | 1175.4 | 0.300000 | 0.304876 | 0.005 | | 1.625 |
| Standard 3 | --- | 2308.0 | 0.600000 | 0.612813 | 0.013 | | 2.136 |
| Standard 4 | --- | 3811.0 | 1.00000 | 1.02145 | 0.021 | | 2.145 |
| Standard 5 | --- | 7355.8 | 2.00000 | 1.98520 | -0.015 | | -0.740 |
| Correlation Coefficient | 0.999790 |  |  |  |  | |  |
| Status | Calibrated |  |  |  |  | |  |
| Curve Type | Linear |  |  |  |  | |  |
| Mn 257.610 Calibration (mg/L) |  |  |  |  |  | |  |
| Standard | Flaqs | Int (c/s) | Std Conc | Calc Conc | Error | | %Error |
| Standard 1 | --- | 5389.0 | 0.100000 | 0.087524 | -0.012 | | -12.476 |
| Standard 2 | --- | 18638.5 | 0.300000 | 0.311572 | 0.0012 | | 3.857 |
| Standard 3 | --- | 35885.4 | 0.600000 | 0.617131 | 0.0017 | | 2.855 |
| Standard 4 | --- | 58249.6 | 1.00000 | 1.01331 | 0.0013 | | 1.331 |
| Standard 5 | --- | 113220.2 | 2.00000 | 1.98709 | -0.013 | | -0.645 |
| Correlation Coefficient | 0.999785 |  |  |  |  | |  |
| Status | Calibrated |  |  |  |  | |  |
| Curve Type | Linear |  |  |  |  | |  |
| Ni 231.604 Calibration (mg/L) |  |  |  |  |  | |  |
| Standard | Flaqs | Int (c/s) | Std Conc | Calc Conc | Error | | %Error |
| Standard 1 | --- | 181.1 | 0.100000 | 0.092228 | -0.008 | | -7.772 |
| Standard 2 | --- | 546.9 | 0.300000 | 0.309716 | 0.010 | | 3.239 |
| Standard 3 | --- | 1048.5 | 0.600000 | 0.607913 | 0.008 | | 1.319 |
| Standard 4 | --- | 1722.5 | 1.00000 | 1.00865 | 0.009 | | 0.865 |
| Standard 5 | --- | 3376.8 | 2.00000 | 1.89223 | -0.008 | | -0.388 |
| Correlation Coefficient | 0.999916 |  |  |  |  | |  |
| Status | Calibrated |  |  |  |  | |  |
| Curve Type | Linear |  |  |  |  | |  |

Table S3. Adsorption kinetics and isotherms equations

| ***Models*** | ***Equations*** |
| --- | --- |
| ***Kinetic*** | |
| Pseudo-First-Order | ${q_{t}= q}_{e}(1-e^{-k_{1}t}$) |
| Pseudo-Second-Order | $q_{t}= \frac{k_{2}q_{e}^{2}t}{1+k_{2}q_{e}t}$ |
| Elovich | $q_{t}=\frac{1}{} ln()+\frac{1}{}lnt$ |
| ***Isotherm*** | |
| Langmuir | $q_{e}= \frac{q_{m}K_{L}C_{e}}{1+K_{L}C_{e}}$ |
| Freundlich | $q_{e}=K_{F} C_{e}^{1/n}$ |
| Temkin | $q_{e}=\frac{RT}{B} {lnAC}_{e}$ |
| k_1_ (min^-1^): *Pseudo-first-order rate constant*  k_2_ (g mg^-1^ min^-1^): *Pseudo-second-order rate constant*  β (g mg^-1^): *Elovich constant*  α (mg g^-1^ min^-1^): *Initial adsorption rate*  q_e_ (mg/g): *Adsorption capacities (equilibrium)*  q_t_ (mg/g): *Adsorption capacities at time t*  q_m_ (mg/g): *Langmuir model maximum adsorption capacity*  K_L_ (L mg^-1^): *Langmuir model adsorption constant*  K_F_ ((mg/g) (L/mg)^1/n^): *Freundlich constant*  n: *Freundlich exponent*  R (J/(mol K)): *Gas constant, 8.314 J/mol·K*  T (K): *Temperature*  B (J mol^-1^): *Temkin constant related to the heat of adsorption*  A (L mg^-1^): *Temkin constant* | |

| 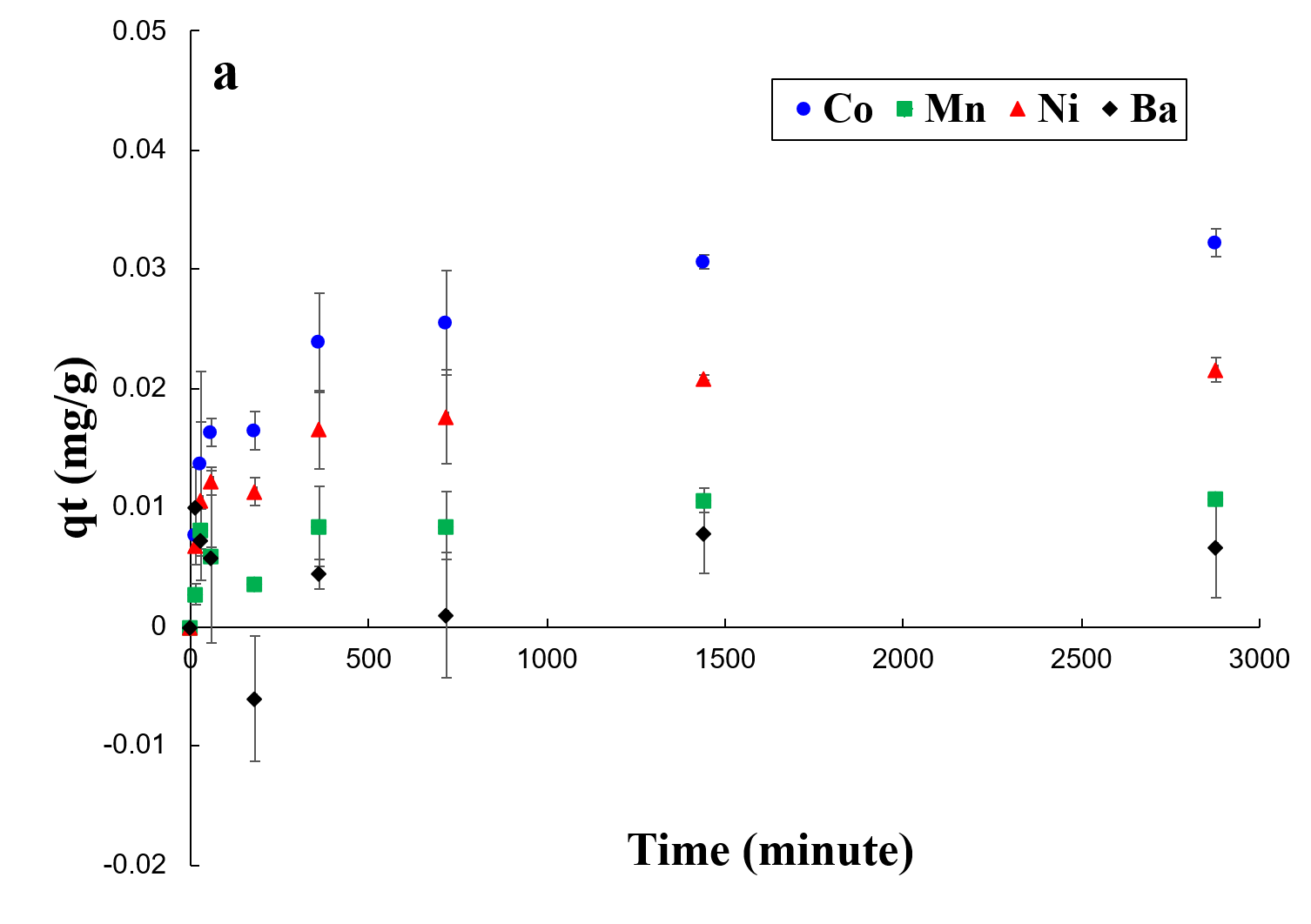 | 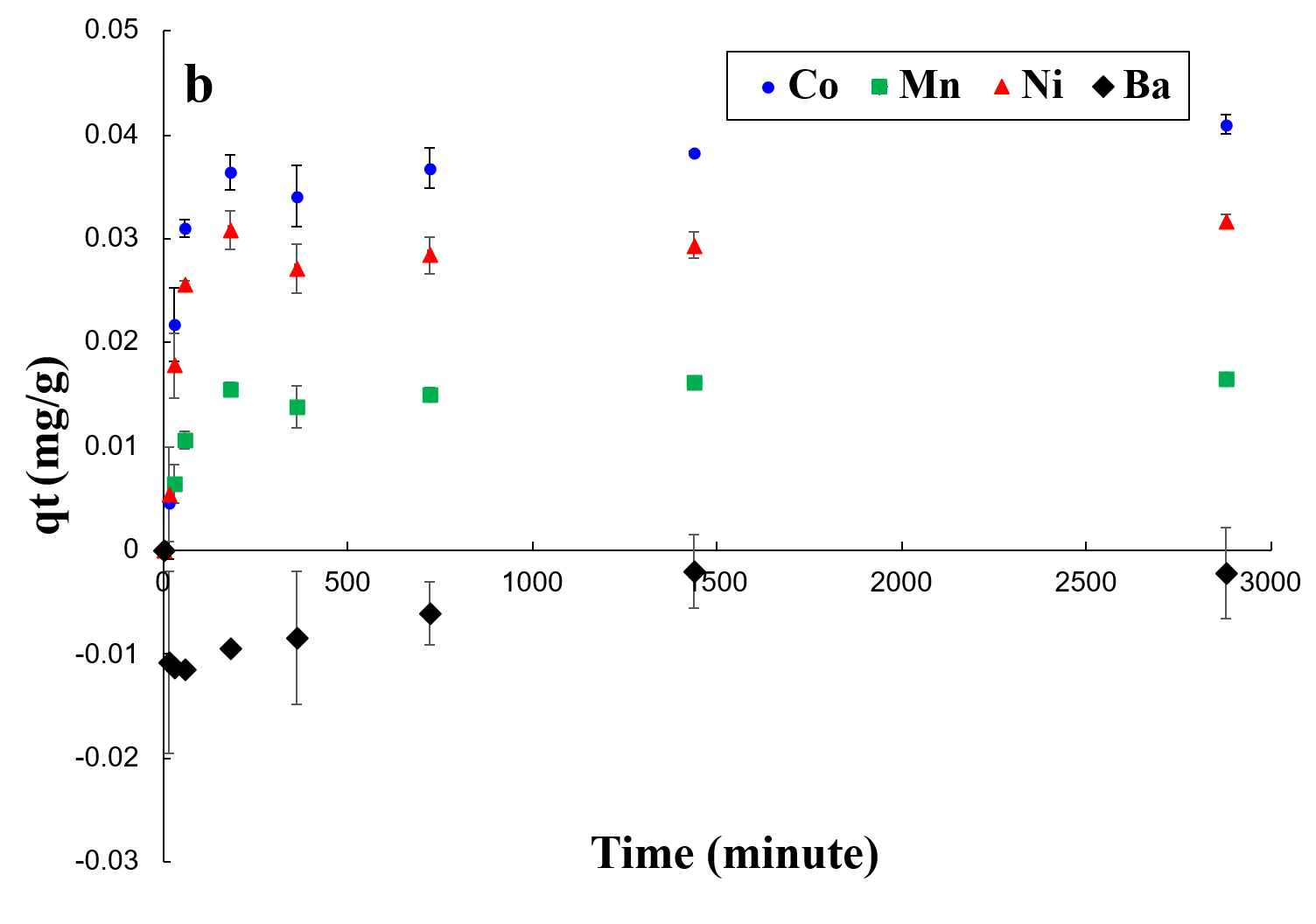 |
| --- | --- |

Figure S1. Kinetic data of a) PVC and b) PP

| 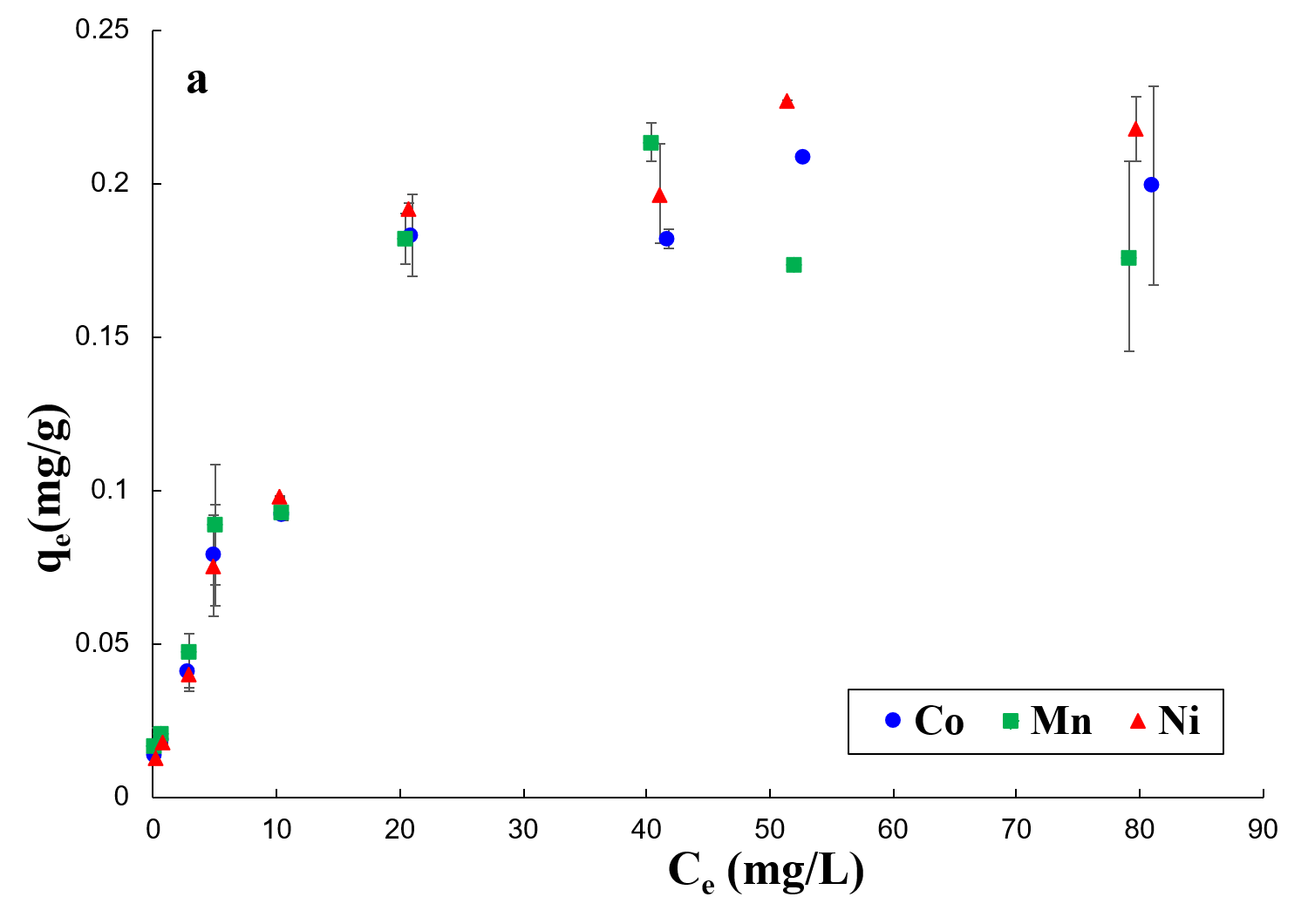 | 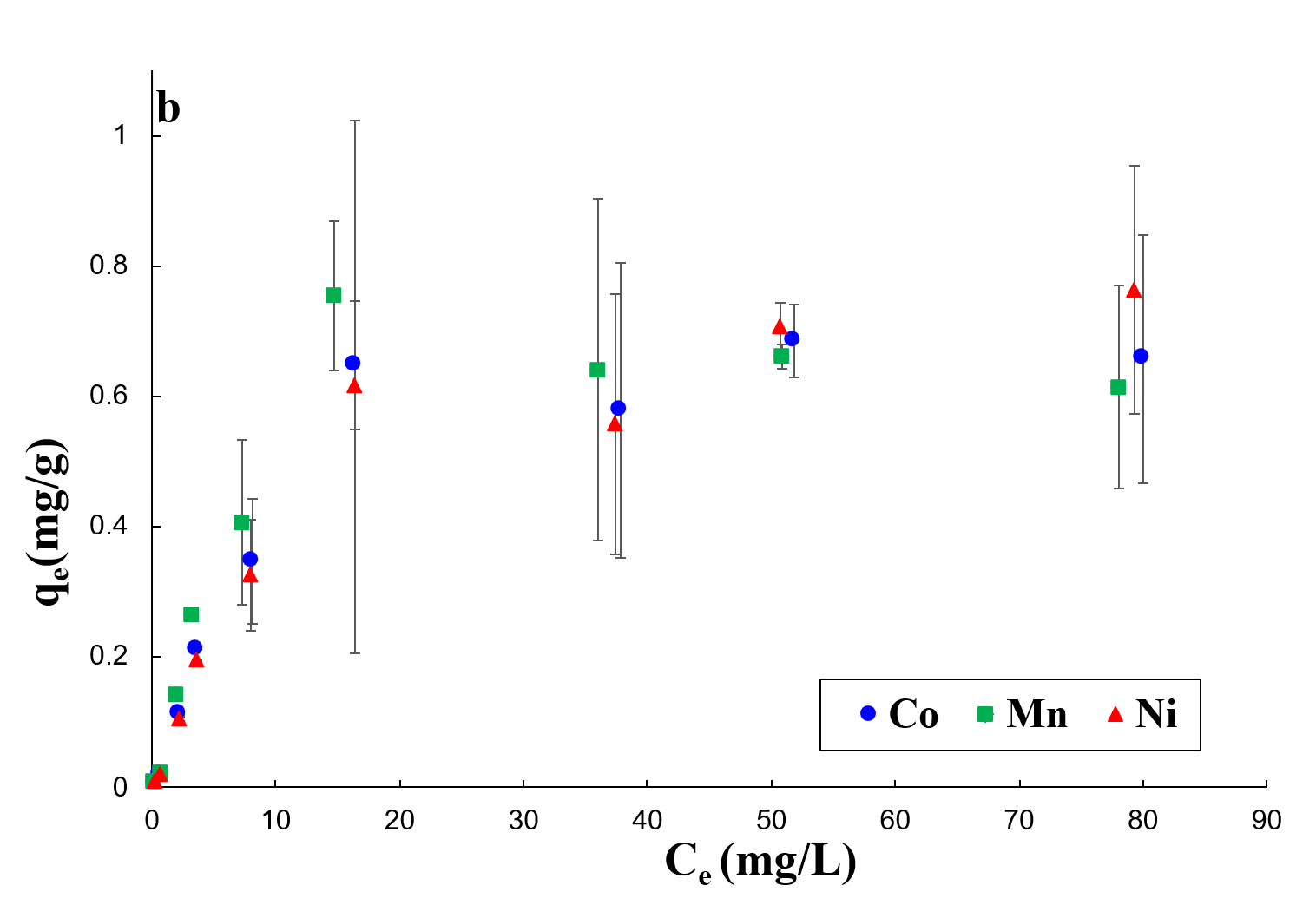 |
| --- | --- |

Figure S2. Adsorption isotherm of a) PVC and b) PP

Table S4. Parameters of non-linear kinetic models for heavy metal adsorption on PVC and PP

| Adsorbent | Metal | Model | k_1_ | k_2_ | α | β | q_e_cal_ | R^2^ | RMSE | HYBRID | MPSD | ARE |  |
| --- | --- | --- | --- | --- | --- | --- | --- | --- | --- | --- | --- | --- | --- |
| PVC | Co | PFO | 0.0160 |  |  |  | 0.0265 | 0.7146 | 0.0043 | 0.131 | 28.144 | 18.925 |  |
| PVC | Co | PSO |  | 0.624 |  |  | 0.0293 | 0.8580 | 0.0030 | 0.069 | 20.728 | 14.666 |  |
| PVC | Co | Elovich |  |  | 0.002 | 217.446 |  | 0.9587 | 0.0016 | 0.023 | 12.832 | 8.831 |  |
| PVC | Ni | PFO | 0.0247 |  |  |  | 0.0177 | 0.6381 | 0.0030 | 0.087 | 26.519 | 17.378 |  |
| PVC | Ni | PSO |  | 1.432 |  |  | 0.0193 | 0.7977 | 0.0022 | 0.052 | 20.937 | 13.606 |  |
| PVC | Ni | Elovich |  |  | 0.003 | 363.895 |  | 0.9362 | 0.0012 | 0.018 | 12.589 | 8.134 |  |
| PVC | Mn | PFO | 0.0437 |  |  |  | 0.0083 | 0.3241 | 0.0023 | 0.143 | 59.004 | 34.613 |  |
| PVC | Mn | PSO |  | 4.823 |  |  | 0.0091 | 0.4264 | 0.0021 | 0.123 | 54.617 | 29.650 |  |
| PVC | Mn | Elovich |  |  | 0.002 | 818.58 |  | 0.5857 | 0.0018 | 0.091 | 47.318 | 26.296 |  |
| PP | Co | PFO | 0.0238 |  |  |  | 0.0377 | 0.9187 | 0.0032 | 0.178 | 58.910 | 23.352 |  |
| PP | Co | PSO |  | 0.767 |  |  | 0.0402 | 0.8871 | 0.0038 | 0.259 | 71.456 | 28.146 |  |
| PP | Co | Elovich |  |  | 0.007 | 183.65 |  | 0.7408 | 0.0057 | 0.541 | 102.353 | 39.809 |  |
| PP | Ni | PFO | 0.0268 |  |  |  | 0.0297 | 0.9268 | 0.0022 | 0.074 | 34.251 | 15.488 |  |
| PP | Ni | PSO |  | 1.192 |  |  | 0.0314 | 0.8644 | 0.0031 | 0.131 | 45.379 | 20.492 |  |
| PP | Ni | Elovich |  |  | 0.011 | 260.549 |  | 0.6713 | 0.0047 | 0.306 | 69.263 | 29.826 |  |
| PP | Mn | PFO | 0.0190 |  |  |  | 0.0155 | 0.9383 | 0.0008 | 0.007 | 7.142 | 5.090 |  |
| PP | Mn | PSO |  | 1.655 |  |  | 0.0165 | 0.9194 | 0.0010 | 0.011 | 9.981 | 6.668 |  |
| PP | Mn | Elovich |  |  | 0.006 | 514.619 |  | 0.7766 | 0.0016 | 0.034 | 19.778 | 10.432 |  |
| *k_1_ (min^-1^), k_2_ (g mg^-1^ min^-1^), α (mg g^-1^ min^-1^), β (g mg^-1^), q_e_ (mg/g)* | | | | | | | | | | | | | |

**Table S5.** Parameters of isothermal models for experimental data regarding the adsorption of heavy metals on PP and PVC.

| Adsorbent | Metal | Model | q_m_ | K_L_ | K_F_ | n | B | A | R^2^ | RMSE | HYBRID | MPSD | ARE |  |
| --- | --- | --- | --- | --- | --- | --- | --- | --- | --- | --- | --- | --- | --- | --- |
| PVC | Co | Langmuir | 0.2414 | 0.0862 |  |  |  |  | 0.9649 | 0.0142 | 0.2947 | 29.8718 | 19.1175 |  |
| PVC | Co | Freundlich |  |  | 0.0408 | 2.5421 |  |  | 0.9141 | 0.0223 | 0.7744 | 50.1529 | 31.4445 |  |
| PVC | Co | Temkin |  |  |  |  | 0.0379 | 2.5491 | 0.8951 | 0.0246 | 2.0545 | 103.8670 | 51.1183 |  |
| PVC | Ni | Langmuir | 0.2692 | 0.0766 |  |  |  |  | 0.9729 | 0.0137 | 0.2591 | 29.2179 | 17.9307 |  |
| PVC | Ni | Freundlich |  |  | 0.0410 | 2.4150 |  |  | 0.9238 | 0.0231 | 0.7952 | 50.9387 | 31.7857 |  |
| PVC | Ni | Temkin |  |  |  |  | 0.0416 | 2.3723 | 0.8922 | 0.0274 | 2.7449 | 123.7060 | 58.6429 |  |
| PVC | Mn | Langmuir | 0.2179 | 0.1210 |  |  |  |  | 0.9215 | 0.0199 | 0.5130 | 36.1918 | 23.5095 |  |
| PVC | Mn | Freundlich |  |  | 0.0488 | 2.9658 |  |  | 0.8494 | 0.0276 | 0.9716 | 48.5635 | 31.8068 |  |
| PVC | Mn | Temkin |  |  |  |  | 0.0320 | 4.7897 | 0.8362 | 0.0288 | 2.0089 | 88.3991 | 50.5494 |  |
| PP | Co | Langmuir | 0.7706 | 0.1188 |  |  |  |  | 0.9538 | 0.0577 | 2.9006 | 126.7350 | 63.1764 |  |
| PP | Co | Freundlich |  |  | 0.1445 | 2.6219 |  |  | 0.8571 | 0.1016 | 24.4747 | 472.6280 | 204.1410 |  |
| PP | Co | Temkin |  |  |  |  | 0.1395 | 1.9637 | 0.9170 | 0.0774 | 17.5773 | 450.2940 | 167.6360 |  |
| PP | Ni | Langmuir | 0.8535 | 0.0865 |  |  |  |  | 0.9650 | 0.0529 | 1.4842 | 71.0229 | 38.0017 |  |
| PP | Ni | Freundlich |  |  | 0.1255 | 2.3229 |  |  | 0.9136 | 0.0831 | 13.4007 | 311.4890 | 141.2440 |  |
| PP | Ni | Temkin |  |  |  |  | 0.1458 | 1.8218 | 0.9217 | 0.0791 | 24.7006 | 499.9320 | 177.0550 |  |
| PP | Mn | Langmuir | 0.7436 | 0.1946 |  |  |  |  | 0.9144 | 0.0802 | 4.1178 | 122.1450 | 60.8975 |  |
| PP | Mn | Freundlich |  |  | 0.1926 | 3.1669 |  |  | 0.7632 | 0.1334 | 32.7443 | 475.4030 | 208.3180 |  |
| PP | Mn | Temkin |  |  |  |  | 0.1315 | 3.1790 | 0.8574 | 0.1036 | 13.3990 | 306.0850 | 133.0660 |  |
| *q_m_ (mg/g), K_L_ (L/mg), K_F_ (mg/g)(L/mg)^1/n^, B (mg/g), A (L/mg)* | | | | | | | | | | | | | | |

Table S6. Comparison of adsorption capacities of microplastics reported in predominantly single-metal systems in the literature

| Polymer | Adsorption capacity (mg/g) | Metals | References |
| --- | --- | --- | --- |
| PP | 0.396 | Pb | (Fan et al., 2021) |
|  | 0.272 | Cu |  |
|  | 0.203 | Cd |  |
|  | 0.194 | Zn |  |
| PE | 1.85 | Cr | (Han et al., 2021) |
| PE | 0.31 | Cu |  |
| PP | 0.45 | Pb |  |
| PAT | 34.68 | Pb | (Yang et al., 2026) |
|  | 29.85 | Cu |  |
|  | 12.31 | Cd |  |
| Nylon | 1.03 | Pb | (Tang et al., 2020) |
| PS | 0.126 | Cu | (Wang et al., 2022b) |
| PET | 0.130 | Cu |  |
| PBT | 1.89 | Cd | (Zhao et al., 2022) |
| PLA | 1.173 | Pb | (Huang et al., 2023) |
| PS | 0.894 | Pb |  |
| PS | 0.162 | Cd | (Wang et al., 2022a) |
| PET | 0.197 | Cd |  |
| Nylon | 0.620 | Ni | (Tang et al., 2021) |
| PP | 1.03 | Pb | (Godoy et al., 2019) |
| PP | 1.04 | Cu |  |
| PVC | 1.06 | Cr |  |
| PVC | 0.467 | Pb |  |
| PS | 0.464 | Co |  |
| *PP: Polypropylene, PE: Polyethylene, PET: Polyethylene terephthalate, PAT: Polyacrylate, PS: Polystyrene, PBT: Polybutylene terephthalate, PLA: Polylactic acid* | | | |

| 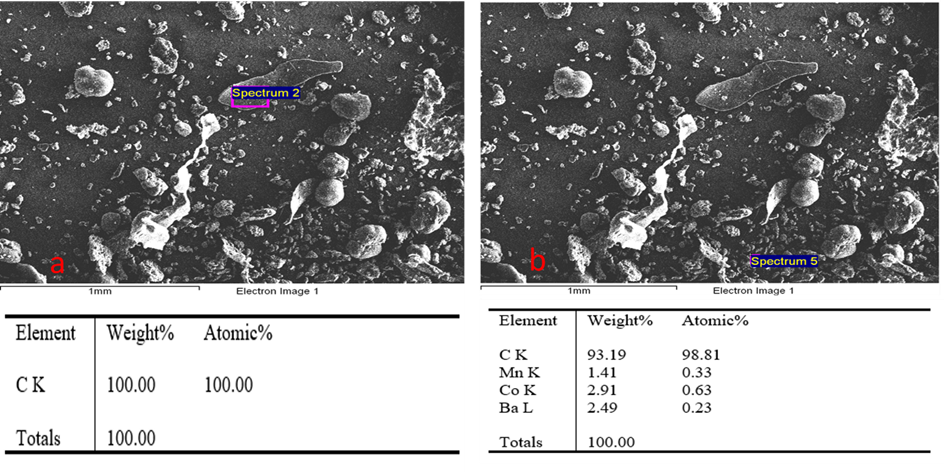  Figure S3. SEM-EDX data for a) pristine PP, b) metal-loaded PP (Ci = 0.5 mg/L) (Magnification: 320X) |
| --- |

| 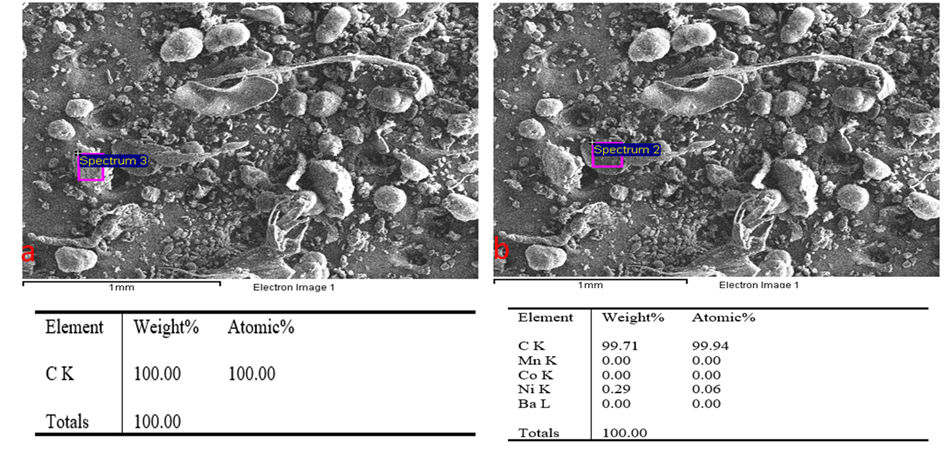  **Figure S4.** SEM-EDX data for a) pristine PP, b) metal-loaded PP (C_i_ = 100 mg/L) (Magnification: 150X) |
| --- |

| 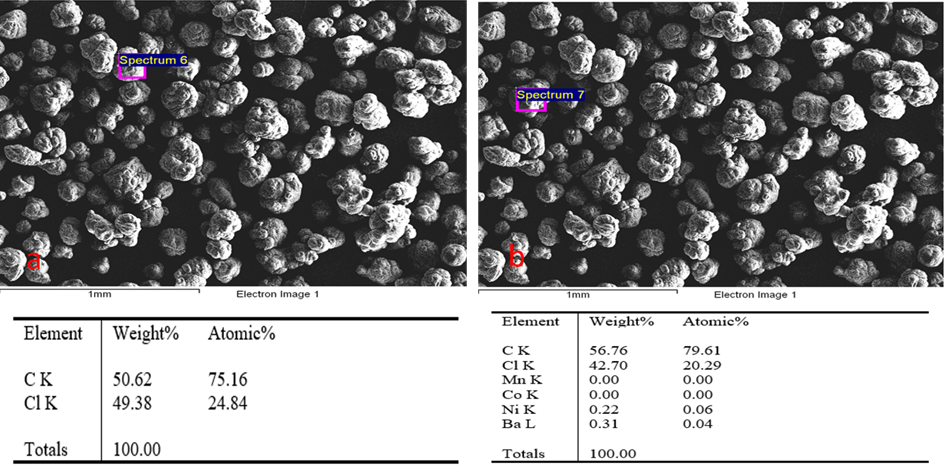  **Figure S5.** SEM-EDX data for a) pristine PVC, b) metal-loaded PVC (C_i_ = 0.5 mg/L) (Magnification: 198X) |
| --- |

| 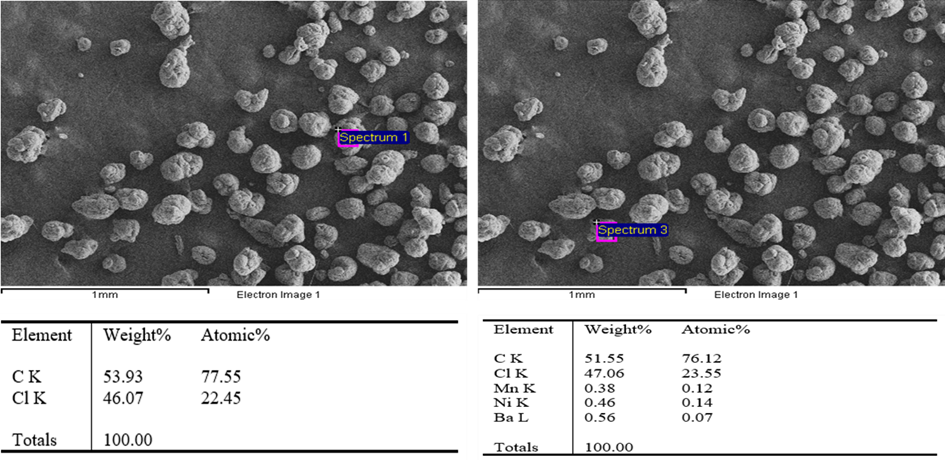  Figure S6. SEM-EDX data for a) pristine PVC, b) metal-loaded PVC (C_i_ = 100 mg/L) (Magnification: 125X) |
| --- |

| 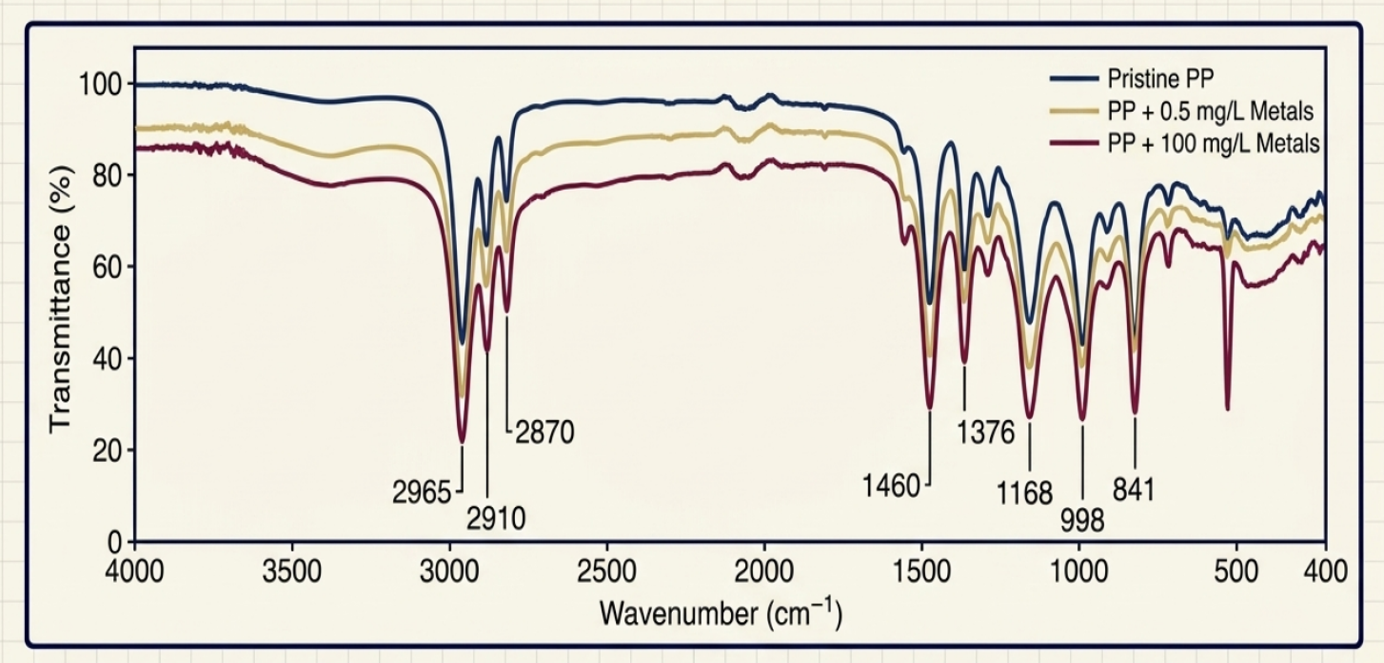  **Figure S7.** FTIR chromatogram of pristine PP and metal-loaded PP (C_i_ = 0.5 mg/L & 100 mg/L) |
| --- |

| 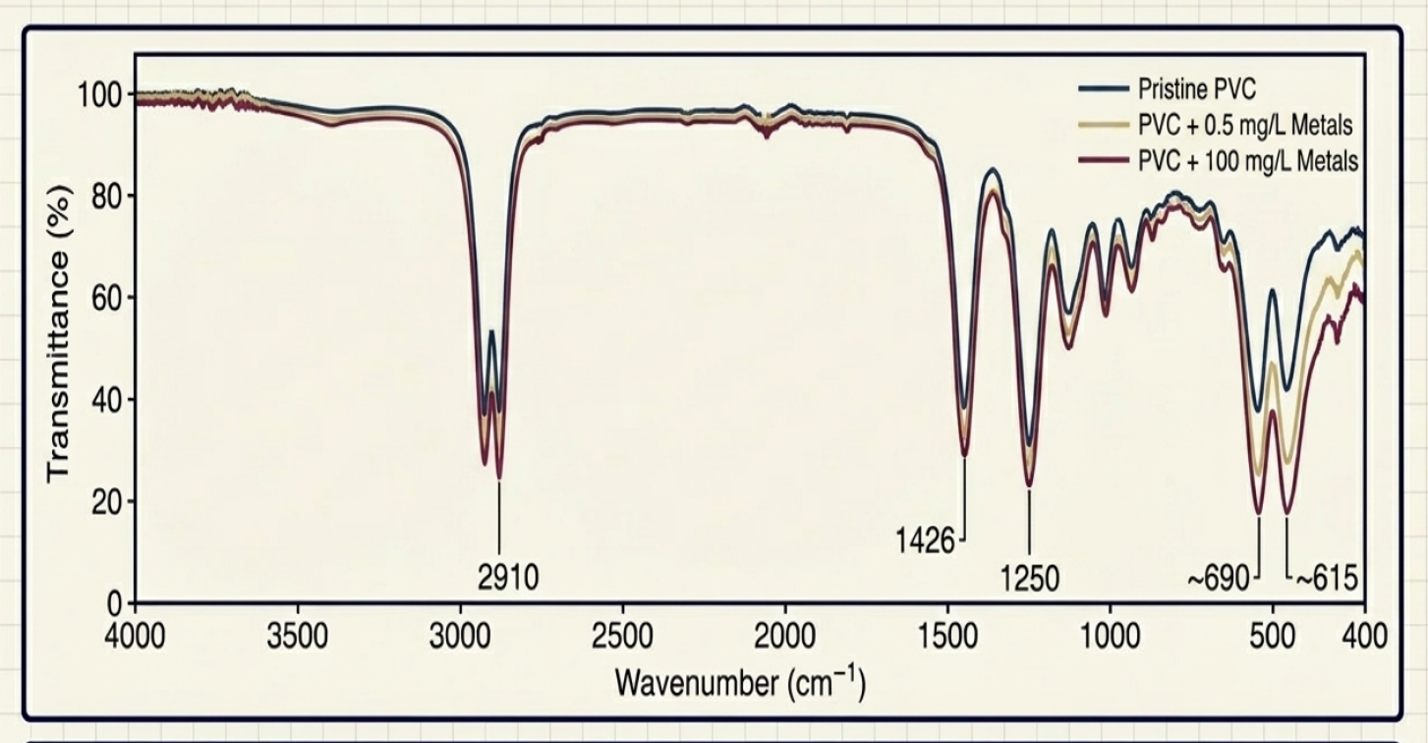  **Figure S8.** FTIR chromatogram of pristine PVC and metal-loaded PVC (C_i_ = 0.5 mg/L & 100 mg/L) |
| --- |

References

Fan, T., Zhao, J., Chen, Y., Wang, M., Wang, X., Wang, S., Chen, X., Lu, A. and Zha, S. 2021. Coexistence and adsorption properties of heavy metals by polypropylene microplastics. Adsorption Science & Technology 2021, 1-12.

Godoy, V., Blázquez, G., Calero, M., Quesada, L. and Martín-Lara, M. 2019. The potential of microplastics as carriers of metals. Environ. Pollut. 255, 113363.

Han, X., Wang, S., Yu, X., Vogt, R.D., Feng, J., Zhai, L., Ma, W., Zhu, L. and Lu, X. 2021. Kinetics and size effects on adsorption of Cu (II), Cr (III), and Pb (II) onto polyethylene, polypropylene, and polyethylene terephthalate microplastic particles. Frontiers in Marine Science 8, 785146.

Huang, W., Deng, J., Liang, J. and Xia, X. 2023. Comparison of lead adsorption on the aged conventional microplastics, biodegradable microplastics and environmentally-relevant tire wear particles. Chem. Eng. J. 460, 141838.

Tang, S., Lin, L., Wang, X., Feng, A. and Yu, A. 2020. Pb (II) uptake onto nylon microplastics: interaction mechanism and adsorption performance. Journal of hazardous materials 386, 121960.

Tang, S., Lin, L., Wang, X., Yu, A. and Sun, X. 2021. Interfacial interactions between collected nylon microplastics and three divalent metal ions (Cu (II), Ni (II), Zn (II)) in aqueous solutions. Journal of Hazardous Materials 403, 123548.

Wang, H., Qiu, C., Song, Y., Bian, S., Wang, Q., Chen, Y. and Fang, C. 2022a. Adsorption of tetracycline and Cd (II) on polystyrene and polyethylene terephthalate microplastics with ultraviolet and hydrogen peroxide aging treatment. Science of The Total Environment 845, 157109.

Wang, X., Zhang, R., Li, Z. and Yan, B. 2022b. Adsorption properties and influencing factors of Cu (II) on polystyrene and polyethylene terephthalate microplastics in seawater. Science of the Total Environment 812, 152573.

Yang, W., Bian, W., Wang, Z., Wu, Q., Lu, F., Qiu, X., Mu, N., Hu, J., Li, Q. and Yang, Y. 2026. Adsorption Characteristics of Heavy Metals onto Functionalized Microplastics. ACS Omega.

Zhao, H., Li, P., Su, F., He, X. and Elumalai, V. 2022. Adsorption behavior of aged polybutylece terephthalate microplastics coexisting with Cd (II)-tetracycline. Chemosphere 301, 134789.
